# A Real-Life Laboratory Setting for Clinical Practice, Education, and Research in Family Systems Care: Protocol for a Transformational Action Research Study

**DOI:** 10.2196/53090

**Published:** 2024-10-30

**Authors:** Evelyn Huber, Erika Harju, Elisabeth Stark, André Fringer, Barbara Preusse-Bleuler

**Affiliations:** 1 Institute of Nursing School of Health Sciences ZHAW Zurich University of Applied Sciences Winterthur Switzerland

**Keywords:** family systems care, family nursing, family health, family well-being, therapeutic conversation, real-life laboratory, transformational action research, case study research, qualitative research, vicarious learning

## Abstract

**Background:**

Burdening health and illness issues such as physical or mental illnesses, accidents, disabilities, and life events such as birth or death influence the health and functioning of families and contribute to the complexity of care and health care costs. Considerable research has confirmed the benefits of a family systems–centered care approach for patients, family caregivers, families, and health care professionals. However, health care professionals face barriers in working with families, such as feeling unprepared. Family systems–centered therapeutic conversations support families’ day-to-day coping, resilience, and health. A family systems care unit (FSCU) was recently established as a real-life laboratory at one of the Swiss Universities of Applied Sciences. In this unit, health care professionals offer therapeutic conversations to families and individual family members to support daily symptom management and functioning, soften suffering, and increase health and well-being. These conversations are observed in real time through a 1-way window by other health care professionals, students, and trainees and are recorded with video for research and education. Little is known about how therapeutic conversations contribute to meaningful changes in burdened families and the benefits of vicarious learning in a real-life laboratory setting for family systems care.

**Objective:**

In this research program, we aim to deepen our understanding of how therapeutic conversations support families and individuals experiencing burdening health and illness issues and how the FSCU laboratory setting supports the learning of students, clinical trainees, and health care professionals.

**Methods:**

Here we apply a transformational action research design, including parallel and subsequent substudies, to advance knowledge and practice in family systems care. Qualitative multiple-case study designs will be used to explore the benefits of therapeutic conversations by analyzing recordings of the therapeutic conversations. The learning processes of students, trainees, and professionals will be investigated with descriptive qualitative study designs based on single and focus group interviews. The data will be analyzed with established coding methods.

**Results:**

Therapeutic conversations have been investigated in 3 single-case studies, each involving a sequence of 3 therapeutic conversation units. Data collection regarding the second research question is planned.

**Conclusions:**

Preliminary results confirm the therapeutic conversations to support families’ coping. This renders the FSCU a setting for ethically sensitive research. This program will not only support the health and well-being of families, but also contribute to relieving the financial and workforce burdens in the health and social care system.

**International Registered Report Identifier (IRRID):**

DERR1-10.2196/53090

## Introduction

### Background

Family functioning and health influence each other [1]. Health-related burdens that affect family functioning and health may arise from physical or mental illnesses, accidents, disabilities, or meaningful life events such as the birth, divorce, or death of family members [2,3]. While supportive collaboration within families improves the behavior and outcomes of chronically ill minor and adult family members, overinvolvement and underinvolvement of families can be harmful [4]. Conversely, taking care of a family member increases the risks of physical and mental symptoms, chronic illnesses [5,6], and chronic sorrow [7] among family caregivers. Family caregivers are also at greater risk of unhealthy behaviors, such as excessive smoking and increased alcohol consumption [6].

Health-related burdens in families contribute to the complexity of care, increasing the demands on nurses and physicians [8,9] and adding to health care costs [10]. Nevertheless, according to literature reviews, health care professionals are optimistic about working with families. Nurses report that the various benefits for patients, families, and nurses outweigh the challenges of working with families [11]. In intensive care, health care professionals have a positive attitude regarding family-centered care and involving families in ward rounds, with physicians and allied health care professionals being more optimistic than nurses [12]. Barriers to working with families in health care are financial pressure, staffing shortage, feeling overburdened or unprepared despite education [2,11,13], lack of communication skills [14], limited intra- and interprofessional collaboration [13], and lack of reimbursement [14]. These factors contribute to the burden of families due to power imbalances between health care professionals and families, families experiencing inconsistent professional practices [13], unmet information needs [14,15], not being involved in discharge planning [11], or lack of care continuity [16].

Family systems care describes an interprofessional health care approach to support families with burdening health issues, recognizing the interactions among individuals, families, significant others, and the larger systems in which they are involved [2]. The term family is used for whom the individuals say it is for them [17], including individuals, their relatives, and significant others [2]. Family systems refer to the family as an entity, its broader social context, and the interactions between the two [2,17]. The literature has widely confirmed the health-related benefits of working with the family rather than the individual patient only for patients, family caregivers, family systems, and health care professionals [12,14,18-25].

One family systems care method is therapeutic conversations aiming to facilitate day-to-day coping and symptom management, relieve suffering, and sustain or regain health and resilience within families and individuals [26]. Therapeutic conversations improve family outcomes such as perceived support from professionals, family functioning, quality of life [27], decision-making, problem-solving, management of the patients’ situations, and recognizing their strengths and needs [11]. Therapeutic conversations have mainly been evaluated in Scandinavian countries in the past years [27].

Therapeutic conversations are based on the Calgary family assessment and interventions model (CFAM/CFIM) [2] and the illness beliefs model (IBM) [3]. The CFAM provides a model to systematically explore a family’s structure, development, functioning, strengths, and needs [2]. The CFIM describes the emotional-cognitive-behavioral responses of health care professionals intended to value the suffering of families and individual family members and reinforce their strengths and resilience [2]. Interventions also comprise recommendations for everyday life and symptom management based on scientific evidence and clinical reasoning [2].

The IBM describes the intersections of health and illness beliefs among individuals, families, and health care professionals, all influenced by broader cultural-societal beliefs [3]. Health and illness beliefs help people to find meaning in health and illness experiences [3]. In therapeutic conversations, both facilitating and constraining health and illness beliefs are explored [3]. Family systems care clinicians help families and individuals to strengthen facilitating beliefs, challenge constraining beliefs, and thus relieve suffering, support healing, and enhance resilience [3].

In Switzerland, family systems care has been taught at different nursing educational levels and established in various health care settings since the beginning of this century [28]. Family systems care has recently expanded to include interprofessional collaboration [29,30]. However, the Swiss Federal Office of Public Health has recognized the need for advancing and coordinating support for families experiencing burdening health and illness issues and establishing educational programs for health care professionals to improve collaboration with these families based on a national research program in 2020 [31]. According to this program, about 1 in 2 family caregivers confirmed a need to talk with health care professionals and a need for continuous, cooperative support [31].

In response to these needs for health care services for families and educational programs for professionals, the School of Health Sciences (SHS) of one Swiss University of Applied Sciences (UAS) launched a family systems care unit (FSCU) as a real-life laboratory. Real-life laboratories are user-centered, cocreative, real-life spaces that foster innovation testing, open innovation [32,33], research cocreation, and knowledge exchange [33]. In the FSCU, therapeutic conversations with families, education and training of health care students and professionals, as well as research in family systems care, are interconnected. The Family Nursing Unit of the University of Calgary [34] and the Center of Excellence in Family Nursing of the University of Montréal [35], both in Canada, and the Family Care Unit at Kalmar University in Sweden [36] were pioneering real-life laboratories for the development and advancement of theoretical knowledge on family systems care. The FSCU fits into the strategy of developing the SHS into a health university, combining interprofessional problem-based and practice-based learning with a contribution to local health care delivery [37].

Although considerable research has been done in family systems care in recent years, a need for further studies was identified in the literature. Regarding family systems care interventions, it is necessary to investigate which mechanisms bring about meaningful changes in families and individuals [20]. Concerning therapeutic conversations, research on positive outcomes, such as family competencies or strengths, is needed in addition to the prevention of adverse health outcomes [27]. Moreover, research on disseminating and implementing family systems care is required [38]. However, health care professionals need to be better prepared, given the barriers to working with families [2,11,13,14]. Finally, other real-life laboratories in health care have mainly been used to test [33] or implement [32] new services. In contrast, we are interested in the benefits of the FSCU real-life laboratory for a deeper understanding of the clinical work with families and individuals and the laboratory’s added value for education and training.

### Aims and Research Questions

Overall, this research program aims to deepen the understanding of the mechanisms of therapeutic conversations and the value of the FSCU laboratory setting for health care professionals and students. Based on this, we will advance the FSCU while contributing to the evidence of therapeutic conversations and the FSCU real-life laboratory as a learning environment.

We will investigate the following research questions: In what way do the therapeutic conversations support burdened families and individuals? In what way does the FSCU laboratory setting support the learning of students, clinical trainees, and health care professionals?

## Methods

### Overall Study Design

In this research program, we apply a transformational action research design as an overall study design [39].

Action research is used to understand practice, introduce innovation, facilitate change, and generate and test local theory [39]. This approach fits well with real-life laboratories’ attributes [32,33]. Unlike previous scientists, Titchen [39] describes the action research process using the metaphor of a tree with the branches representing parallel, overlapping, and subsequent action research cycles relating to each other rather than a sequence of linear subsequent action research cycles. Each cycle consists of three phases: (1) planning, (2) acting and observing, and (3) reflecting and revising the processes under development [39]. The action research process of this research program will be broken into substudies, building on and complementing each other flexibly to remain adaptive to unexpected changes while working towards achieving the study aims [39]. The substudies are (1) planned, (2) performed, and (3) reflected on by the researchers and clinicians to decide on changes in the work of the FSCU and additional substudies needed.

Transformational action research is suitable for transforming the practice as the aim and means of the research program, as well as for the personal transformation of all those involved in the program by enabling human flourishing through the facilitation of personal growth and development [39].

### Study Setting and Intervention

The study setting is the FSCU as part of the SHS of one Swiss UAS.

The therapeutic conversations based on the CFAM, the CFIM, and the IBM have been chosen as interventions because of their longstanding, international use in health care and their purpose of strengthening the families’ resilience, everyday symptom management, and coping with health-related suffering, challenges, and needs [2,27]. The therapeutic conversations are moderated by a health care professional trained at the Master of Science level who specialized or specializes in family systems care. These health care professionals work in tandem (ie, a clinical expert and a trainee), one leading the therapeutic conversation and one available for reflection and mutual professional support. Since the data from the therapeutic conversations are used for research and education, the service is free of charge for the families.

Regarding education and training, the FSCU provides a clinical and research learning environment for health care students, postmaster trainees in family systems care, external partners, and the FSCU experts and researchers. The FSCU comprises 2 rooms with a 1-way window between them. It is equipped with an audio transmission system and video-recording technology. The therapeutic conversations take place in one of the 2 rooms. They are observed from the adjacent room through the 1-way window by the tandem partner, further clinical trainees and experts, health care students, researchers, or other guests such as health care professionals from external partner institutions. The therapeutic conversations are prepared, reflected on, and evaluated in presession and postsession discussions to ensure the quality of the therapeutic conversations and the reflection and learning processes of the present persons. In addition, post-master trainees conduct family conversations with live supervision from experts and peers. Furthermore, Master of Science and doctoral students accomplish their theses within this research program while advancing their clinical skills. Finally, selected video-recorded therapeutic conversations are used in classroom teaching of the UAS.

### Populations

#### Overview

In this research program, we target two study populations: (1) families and individuals experiencing burdening health and illness issues and (2) students, trainees, and health care professionals.

#### Families or Individuals

We target families or individuals with any health conditions or life events of one or several family members that burden their functioning and health. As of July 2024, a total of 57 families of various age groups, family compositions, and life phases used the service of the FSCU. Examples were families with minors with a family member affected by a mental illness, such as anorexia in an adolescent or schizophrenia in a parent, or a physical illness, such as cancer, chronic pain, or fatigue in a parent. Other families were severely affected by COVID-19 due to the early death of a parent or post–COVID-19 condition in an adult sibling. Furthermore, families with a child with intellectual disability, couples who have experienced an acute myocardial infarction or are living with multiple sclerosis, and a family with an older family member in preparation for assisted suicide used the FSCU service. Often more than 1 family member is affected by a health condition that can be related to or independent of each other.

### Study Design

We investigate therapeutic conversations using qualitative multiple-case study designs [40-42]. Case study research aims to reach a nuanced understanding of a case or multiple cases by finding patterns, insights, or concepts in the cases [41]. In multiple-case study designs, various single cases are compared and contrasted regarding similarities and differences [41]. Naturalistic, analytical generalizations, rather than statistical generalizations, can be derived from multiple-case studies to advance existing theoretical propositions, uncover new ones [41], learn from particular cases, and transfer and apply the learnings to populations of similar contexts [42].

In case-study research, cases are designated by defining the bounded systems constituting the casing [41,43]. In this research program, we define a case as a family, a unit of family members (ie, a couple or parents), or an individual participating in FSCU therapeutic conversations. The temporal boundaries of a case are defined as the period between the first contact of a family or individual with the FSCU and the end of the therapeutic conversations. The spatial boundaries are defined by the FSCU laboratory setting, exceptionally web-based, using a videoconference tool, or in a family’s home. The boundaries by activity are therapeutic conversation units. One therapeutic conversation unit includes a presession, a therapeutic conversation, and a postsession.

### Recruitment

We recruit families or individuals with 3 procedures (Textbox 1).

**Procedures for recruiting families or individuals.**

- Families or individuals contact the family systems care unit (FSCU) as an advisory service via the Internet contact form, e-mail, or telephone.
- Other health care providers recommend the FSCU to families.
- We recruit families or individuals purposefully through health and social care organizations. We will ask these organizations to look for families interested in participating in a case study, initiate contact, and transfer the contact data to the clinical experts for further explanation and organization of an appointment.

### Inclusion and Exclusion Criteria

We will include families or individuals in the study regardless of their financial situation, cultural background, age of family members, or gender diversity in families. We will include families, units of family members, or individuals meeting the following criteria (Textbox 2).

We will exclude families, units of family members, or individuals meeting the following criteria (Textbox 3).

**Inclusion criteria.**

- If they are making use of the therapeutic conversations for the first time
- If they are taking part in one single or a sequence of therapeutic conversation appointments
- If these appointments are prepared and evaluated by a presession and postsession with the clinical team
- If the therapeutic conversations, the presessions and postsessions are recorded with sound or sound and video.

**Exclusion criteria.**

- If they do not indicate a burden at all during the therapeutic conversations
- If they previously used the family systems care unit (FSCU) service and the therapeutic conversations were regarded as finished.

### Sample Size and Sampling

Yin [41] recommended at least 4-6 cases in multiple-case study designs using a theoretical replication strategy if heterogeneous or even contradictory findings emerge from single cases. Theoretical replication means selecting new cases according to essential findings from previous ones [41]. Sandelowski [43] argues for small sample sizes representing a variety of characteristics of the target population and studying them intensively. Creswell and Poth [42] propose a maximum of 4-5 cases to identify themes across and between the single cases.

Since the families visiting the FSCU are heterogeneous in situations, health problems, and family compositions, we plan for a 2-step theoretical replication strategy [41]. Thus, we will group 3-6 cases into a first multiple-case study and add further multiple-case studies if needed to answer research question one, depending on whether the data will allow for analytical generalizations [41]. This proceeding is in line with the action research approach because it supports the application of action research cycles, including the reflection and adaptation of the FSCU practice, before starting a new action research cycle.

### Data Collection

We apply data triangulation, which is typical in case-study research [41]. We collect qualitative data for each case from the therapeutic conversations, the team’s presessions and postsessions, video or audio recordings, nonparticipatory observations through the 1-way window, and the clinical team’s workbook notes.

For video and audio recordings, we simultaneously use the FSCU’s permanently installed video and audio system and, as backup, additional recording tools offered within the secure authentication method of the UAS or other mobile camera systems, allowing safe data recording.

In terms of observations, we take nonstructured notes on observations regarding the aspects that were discussed in the presession based on the theoretical models of this study. Such aspects are a family’s strengths and needs on the cognitive, emotional, and behavioral levels of family functioning [2], a family’s health and illness beliefs [3], and moments indicating meaningful changes during the therapeutic conversation, such as emotional reactions of family members [2,3].

In the clinical team’s workbook, the geno-ecogram of the closer and wider family’s structure and relationships [2], important information, such as medical diagnoses of family members, and a synthesis of the presessions, the therapeutic conversations, and the postsessions based on a semistructured list of topics are summarized. With regard to the presessions, such topics are information about the family’s concerns to be discussed, evidence-based information on relevant topics, for example, on medical diagnoses and treatments or health and social insurance issues, hypotheses regarding the family’s strengths, health and illness beliefs, and needs, or red flags the clinician must be aware of, such as sensitive topics around a family’s suffering. Topics of the therapeutic conversations to be noted are the family’s concerns for the current conversation, the topics discussed, the feedback of the family regarding the conversation, and decisions on how to proceed. Regarding the postsession, moments of surprise, success, difficulty, and open questions for subsequent therapeutic conversations are summarized.

### Data Analyses

#### Overview

All recordings are transcribed verbatim, either by students or external transcribers, as soon as participants are included in a substudy of this research program. We will use the transcripts and video recordings for the data analyses.

Following Yin [41] and Sandelowski [43], we will analyze the data in 3 steps, focusing on the case level (Textbox 4).

For inductive analyses, we will specify the methods for every substudy using interpretive description [44], content analysis, or thematic analysis [45]. These methods use coding methods to find, organize, and describe themes, patterns, and concepts meaningful for clinical practice in the data [44-46]. We will use various methods because of the real-life laboratory setting, which allows master of science and doctoral students working on case studies to consider suitable data analysis methods.

For deductive comparisons within a sequence of therapeutic conversation units and across cases, we will rely on the theoretical assumptions of the CFAM/CFIM [2] and the IBM [3]. Following these models, we will look for similarities and differences across the cases in the cognitive, emotional, and behavioral domains of the therapeutic conversations [2] and regarding facilitating and constraining health and illness beliefs of the families [3].

Following the theoretical replication strategy described by Yin [41], we will perform recruitment, data collection, and data analyses in an overlapping process. This process will enable us to include new cases based on the preliminary results and unanswered questions of the previous cases and thus reach analytical generalization [41]. We will use the software MAXQdA [47] to analyze qualitative data.

**Three steps to analyze data.**

- We will analyze each therapeutic conversation unit for each case inductively and in-depth (first step of within-case analysis).
- We will inductively and deductively compare the sequence of therapeutic conversation units for each case (second step of within-case analysis).
- We will compare multiple cases for similarities and differences using an inductive-deductive approach (cross-case analysis).

#### Students, Trainees, and Health Care Professionals

We further target SHS students of different levels of education, such as bachelor of science, master of science, programs for advanced education, postmaster programs for advanced practitioners for trainees in family systems care, and doctoral students.

Finally, we target health care professionals, such as the FSCU’s clinical experts or external guests.

### Study Design

Descriptive qualitative research designs are used to investigate the learning experiences of students, trainees, and health care professionals [45,46,48]. These designs investigate research questions emerging from practice, such as educational practice, to provide useful insights for the practice in question [44-46].

### Recruitment

Students, trainees, and health care professionals are recruited by direct contact with a researcher or one of the FSCU coheads.

### Inclusion and Exclusion Criteria

We include or exclude students, trainees, and health care professionals as per Textbox 5.

**Inclusion and exclusion criteria.**

**Inclusion criteria**

- If they observe one or more therapeutic conversations through the 1-way window of the family systems care unit (FSCU) and participate in the presessions and postsessions, or
- If they take on an active role as a clinical team member in one or more therapeutic conversation units, or
- If they work with FSCU sound or video-recorded data of one or more therapeutic conversation units to complete their Master of Science or doctoral theses, or
- If they participate in classroom teaching based on video recorded material from the FSCU.

**Exclusion criteria**

- If they do not get in contact with real-life and/or video recorded data from one or more therapeutic conversation units.

### Sample Size and Sampling

In terms of sample size, the number of participants varies from 8 to 50 in descriptive qualitative interview studies [49]. According to Malterud et al’s [50] concept of information power, samples with ample information power need small sample sizes, and vice versa [50]. Thus, we estimate a medium sample size of 20 participants in a first action research cycle due to the narrow and specific research question on the one side (indicating high information power), the lack of existing theory, the low quality of dialogue expected due to the limited content to be discussed, and the focus of analysis across interview participants on the other side (indicating a larger sample size needed) [50]. If required, it is essential to adapt the sample size during the research process [50]. We will reflect on this during the action research process.

We choose a maximum variation sampling strategy typical in descriptive qualitative research [46] to gain information about participants with various backgrounds in terms of education level, health care practice experience, age, and gender, as well as having observed therapeutic conversations at the FSCU or worked with video material in classroom settings.

### Data Collection

We will collect qualitative data from students, trainees, and health care professionals in single or focus group interviews based on semi-structured interview guides. These interviews will start with the question: “Can you tell me what it was like for you to participate in this therapeutic conversation at the FSCU/in this classroom teaching working with the video of a therapeutic conversation?” Depending on the interview proceeding, prompts will be used, such as: “Can you tell me more about this particular sequence of the conversation you mentioned?” Finally, more specific questions will be asked, such as: “How was it for you if the expert asked your considerations during the postsession?”

The interviews will take place in a quiet room at the SHS, exceptionally web-based, using a videoconference tool within about 4 weeks after observing a therapeutic conversation or the classroom teaching with video material of a therapeutic conversation. They will be recorded with audio or video.

### Data Analyses

Students or external transcribers transcribe all recordings verbatim as soon as participants are included in a substudy of this research program.

The qualitative data will be analyzed inductively using interpretive description [44], content analysis, or thematic analysis [45], depending on the considerations of the researchers and students involved in the data analysis. We will use the software MAXQdA [47] to analyze qualitative data.

### Ethical Considerations

#### Ethical Approval and Informed Consent

This research program does not fall under Swiss legislation regarding human research, as confirmed by the Cantonal Ethics Committee on the December 14, 2021 (waiver no. Req-2021-01424). Nevertheless, we will take measures to ensure good clinical practice guidelines and follow the Swiss legislation on data protection. First, we received ethical approval for this study protocol from the internal ethical commission of the UAS on the October 18, 2024 (EA-ZHAW 2024-027-G). Second, the researchers of this research program are trained in good clinical practice. Third, we ask all participants involved in the FSCU therapeutic conversation units and individual or focus group interviews to provide informed consent.

The informed consent form includes a range of levels of agreement, from agreeing to recordings to observation to providing the data for research and education. If someone does not agree to video recordings and observation, we offer an adapted solution, such as audio recording only, to ensure data collection for research.

Participation in this research program is voluntary. All participants can always withdraw from participation in the research program without giving reasons and without experiencing any disadvantages due to their withdrawal. Data used in ongoing substudies will then be used to finish this substudy. Subsequently, the data will be electronically sealed with a password known only to the 2 coheads of the FSCU.

All health care professionals, students, transcribers, and other persons with access to the clinical data of families and individuals or single or focus group interviews within their activities must sign a confidentiality agreement form.

#### Data Management

All personal data, video and audio recordings, transcripts, and professional documentation are stored securely. We store all electronic data on a restricted UAS server. All transcripts are anonymized. If AI-based tools for transcription or data analyses will be used, their alignment to Swiss data protection laws will be carefully checked.

Each family is given a number and an alias for internal communication. The 2 coheads of the FSCU have access to the key file, which connects personal data with the family number and alias. This password-secured key file is saved on a restricted UAS server.

The video and audio recordings are transferred by a clinical team member or one of the 2 coheads of the FSCU to the secure data storage via secure authentication methods within a maximum of 48 hours and then deleted on the source tool. The secure data storage is structured according to families. The clinical team has access to all family folders; researchers, trainees, and involved students have access only to the documents of the families with whom they work. Data material collected by students in connection with master theses is kept in a single secure restricted folder per master thesis, to which only students and the responsible research supervisors within the FSCU team have access.

Paper documents are saved in a locked cupboard in a locked room inside the FSCU as long as a family or individual is visiting or a substudy is proceeding. The rooms, including the video system’s server room, are in a part of the SHS with an individual badge access system and a reception desk staffed on working days. When a series of therapeutic conversation units or one of the substudies is finished, the paper documents are stored in a locked research archive with restricted entrance regulations.

### By Swiss Law, All Data Will Be Stored for 20 Years at the UAS

#### Risks and Benefits

The double nature of the FSCU, as a service for families and individuals and as a laboratory for research and education, bears 3 main risks for families and individuals. First, the families and individuals, being in a vulnerable situation due to their burdening health and illness issues and maybe even in urgent need of help, are asked to consent to the sharing of their experiences and thoughts with an additional “audience,” immediately in the observation and later by allowing the use of the recordings for research and educational purposes. This may add to the pressure on those families or individuals. Second, they often live in the region of the SHS and may previously be known to professionals or students if their data are used in SHS classroom courses. Third, they are in a situation of dependency due to their need for help and lack of such services.

For these reasons, and beyond the affirmation of anonymity, it is crucial to assure confidentiality regarding sensitive and personal information by creating trusting relationships in addition to the informed consent process and written confidentiality agreements [51]. With these risks in mind, the clinical expert carefully explains the study information to the families and individuals, including the benefits for them, the FSCU’s purposes in terms of research and education, and the UAS data protection and confidentiality measures, before asking them to sign the informed consent form. In classroom education, transcript excerpts will be anonymized and carefully chosen so as not to intrude on anyone’s anonymity. In using video or sound recordings in education, one of the coheads will carefully select sequences of therapeutic conversations containing no personal features. This precaution applies over and above the consent of the family or individual to use their recordings for this purpose.

If families or individual family members deny recording and observing therapeutic conversations at all, ethical weighing is necessary. The benefits of therapeutic conversations are well known, making it ethically problematic to refuse burdened families or individuals. In such cases, individual solutions will be sought, such as helping to find another suitable professional service.

Considering the dependency of families, 2 measures are taken within the clinical routines. First, the visiting families and individuals are asked towards the end of every therapeutic conversation session how they perceive it, how they want to proceed, and whether they want to change anything in the next session. Second, red flags the clinical expert must be aware of are routinely discussed in the postsessions. Furthermore, therapeutic conversations are not part of the medical treatment of families and individuals, reducing the risk of dependency.

Regarding benefits, the FSCU provides a professional and sensitive data collection setting. The data of the families and individuals are collected in conversation situations where they receive emotional and practical support from the family systems care expert to lower their burdens. Furthermore, they are extended the opportunity for additional therapeutic conversations if desired.

In single and focus group interviews with students, trainees, and health care professionals, there is a risk of harm when participants are open to others and of dependency regarding education or working situation. Single and focus group interviews will be performed by experienced interviewers or by novice interviewers who have received training and will receive supervision. The interviewers will assure confidentiality orally and in writing as part of the study information. We judge that the benefits of sharing experiences and considerations outweigh this population’s potential risks.

#### Data Quality Assurance

An experienced senior researcher leads the research program, which is staffed with 3 postdoctoral researchers. The FSCU was established and is comanaged by an experienced clinical family systems care expert in close contact with and relying on Canadian pioneers in this kind of work [34,35]. All researchers and clinicians are trained and experienced at the master of science or doctoral level in nursing, midwifery, or family medicine, and most of them also teach in higher education.

We will assure data quality by adhering to guidelines for evaluating case studies and qualitative research as appropriate to the methods used in each substudy. Above all, we will engage in reflexive thinking [52] within the research and clinical team, and between researchers, clinical experts, trainees, and students.

## Results

As of the July of 2024, a total of 3 single-case studies examined how therapeutic conversations supported families. Each investigated a sequence of 3 therapeutic conversation units. The manuscripts of these substudies are being prepared for publication. Data collection and analysis of further substudies are ongoing.

The data collection with regard to the second research question needs to be planned.

## Discussion

### Anticipated Findings

The results of our first single-case studies confirm the usability of the FSCU data and the case study approach to answer how therapeutic conversations support burdened families and individuals. The results confirm that therapeutic conversations support families in the way that they strengthen the families regarding coping with their longstanding, burdening, and complex situations, even if the challenges cannot be resolved. This is in line with a recent literature review, where 12 out of 14 studies approved therapeutic conversations to be effective for increasing perceived family support in various populations [27]. Previous qualitative studies showed that families developed new perspectives on their situations, felt facilitated in sharing their individual beliefs, and strengthened the family members’ relations after participating in therapeutic conversations [27].

With this research program, we also intend to investigate the benefit of the FSCU as a real-life laboratory and learning environment for students, trainees, and health care professionals. Learning experiences among students, trainees, and professionals in the FSCU have anecdotally been told to improve traditional learning methods. In Canada, this setting initiated a learning process for nurses continuing in their clinical work [35]. Learning from each other’s experiences is called vicarious learning [53], which is seen as a fundamental human skill and a complex task involving various cognitive processes and brain systems [54]. Vicarious learning occurs when valuing the experiences of others, feeling engaged in the situation, trying to understand it, and in discursive reflection [53]. To the best of our knowledge, evidence regarding the benefits of real-life laboratory settings for vicarious learning is scarce.

In qualitative research, there is always a risk of a hierarchical power imbalance due to the interviewees revealing themselves without receiving information regarding their health and illness issues [42,51]. They are then left alone with issues that may have arisen in the interview. This is particularly critical for families and individuals in vulnerable situations, even more so if families include their minor children or sick family members [51]. Using participatory methods can reduce power imbalances [51]. This research program addresses this by the clinical experts trained in working with participatory, non-hierarchical conversation methods. In addition, families and individuals determine the topics of the conversations and receive professional support regarding the day-to-day coping of their situations. They are also offered additional appointments in the FSCU if needed. This makes the FSCU a setting for ethically sensitive and justifiable research.

Furthermore, the FSCU, as a laboratory setting, reduces power imbalances among clinicians, researchers, and students and supports participatory collaboration and mutual learning. On the one hand, the clinical experts open the therapeutic conversation units to researchers and students by involving them as passive observers in the therapeutic conversations and as active contributors in the presessions and postsessions. On the other hand, clinical experts become coresearchers in action research, which has led to the term practitioner-researchers [39]. Accordingly, they receive an active role in contributing to the generation of scientific knowledge and, at the same time, in investigating their expertise, which often remains hidden in health care professions [39].

### Limitations

The research program is prone to bias due to dependence between families or family members and clinicians, mentors and trainees, and researchers and students. Selection bias cannot be ruled out for the families and individuals attending the FSCU. Individuals with lower incomes may visit the FSCU since the service provided is free of charge. Due to the study designs, conclusions do not allow for statements regarding causality. However, case study and qualitative research approaches aim to gain deeper insights into the meaning individuals or groups assign to phenomena in their everyday lives [41,42]. Quantitative and mixed methods studies [40,55] are also planned in the future.

### Conclusions

The FSCU data and the case-study approach show that therapeutic conversations support families and are beneficial for their coping. This research program aligns with the need for further research in family systems care, as recently identified in a comprehensive umbrella review [56]. In particular, there is a need to understand the mechanisms leading to meaningful changes in families [20] and further studies on the outcomes of therapeutic conversations [27]. We contribute to these gaps by applying a transformational action research program and non-hierarchical conversation methods. This makes the FSCU a setting for ethically sensitive and justifiable research. This research will help to alleviate family and individual suffering, promote their health and well-being, and thus, indirectly, relieve the financial and workforce burdens in the health and social care system.

## Abbreviations

CFAM: Calgary family assessment model
CFIM: Calgary family interventions model
FSCU: family systems care unit
IBM: illness beliefs model
SHS: School of Health Sciences
UAS: University of Applied Sciences

## Data Availability

The datasets generated or analyzed during this research program are not publicly available due to their high sensitivity and the vulnerability of the participating families. However, data on the learning experiences of students, trainees, and health care professionals are available from the corresponding author upon reasonable request.

## References

[1] Rolland JS (2017). Neurocognitive impairment: addressing couple and family challenges. Fam Process.

[2] Shajani Z, Snell D (2023). Wright & Leahey's Nurses and Families: A Guide to Family Assessment and Intervention, 8th Edition.

[3] Wright LM, Bell JM (2021). Illness Beliefs: The Heart of Healing in Families & Individuals, 3rd Edition.

[4] Martire LM, Helgeson VS (2017). Close relationships and the management of chronic illness: associations and interventions. Am Psychol.

[5] Smith L, Onwumere J, Craig T, McManus S, Bebbington P, Kuipers E (2014). Mental and physical illness in caregivers: results from an English national survey sample. Br J Psychiatry.

[6] Stacey AF, Gill TK, Price K, Taylor AW (2018). Differences in risk factors and chronic conditions between informal (family) carers and non-carers using a population-based cross-sectional survey in South Australia. BMJ Open.

[7] Coughlin MB, Sethares KA (2017). Chronic sorrow in parents of children with a chronic illness or cisability: an integrative literature review. J Pediatr Nurs.

[8] Yoshida S, Matsushima M, Wakabayashi H, Mutai R, Sugiyama Y, Yodoshi T, Horiguchi R, Watanabe T, Fujinuma Y (2019). Correlation of patient complexity with the burden for health-related professions, and differences in the burden between the professions at a Japanese regional hospital: a prospective cohort study. BMJ Open.

[9] Huber E, Kleinknecht-Dolf M, Kugler C, Spirig R (2021). Patient-related complexity of nursing care in acute care hospitals - an updated concept. Scand J Caring Sci.

[10] Sugiyama Y, Mutai R, Matsushima M (2023). Association between patient complexity and healthcare costs in primary care on a Japanese island: a cross-sectional study. BMJ Open.

[11] Mileski M, McClay R, Heinemann K, Dray G (2022). Efficacy of the use of the Calgary Family Intervention Model in bedside nursing education: a systematic review. J Multidiscip Healthc.

[12] Kydonaki K, Takashima M, Mitchell M (2021). Family ward rounds in intensive care: an integrative review of the literature. Int J Nurs Stud.

[13] Naef R, Brysiewicz P, Mc Andrew NS, Beierwaltes P, Chiang V, Clisbee D, de Beer J, Honda J, Kakazu S, Nagl-Cupal M, Price AM, Richardson S, Richardson A, Tehan T, Towell-Barnard A, Eggenberger S (2021). Intensive care nurse-family engagement from a global perspective: a qualitative multi-site exploration. Intensive Crit Care Nurs.

[14] Glajchen M, Goehring A, Johns H, Portenoy RK (2022). Family meetings in palliative care: benefits and barriers. Curr Treat Options Oncol.

[15] Lange S, Mȩdrzycka-Da Browska W, Friganović A, Religa D, Krupa S (2022). Family experiences and attitudes toward care of ICU patients with delirium: a scoping review. Front Public Health.

[16] Kläusler-Troxler M, Petry H, Lanter R, Naef R (2019). Implementing family systems nursing through a participatory, circular knowledge-to-action research approach in women’s health. Int Pract Dev J.

[17] Kaakinen JR, Kaakinen JR, Coehlo DP, Steele R, Robinson M (2018). Family Health Care Nursing. Family Health Care Nursing. Theory, Practice, and Research.

[18] Chartrand J, Shea B, Hutton B, Dingwall O, Kakkar A, Chartrand M, Poulin A, Backman C (2023). Patient- and family-centred care transition interventions for adults: a systematic review and meta-analysis of RCTs. Int J Qual Health Care.

[19] Chesla CA (2010). Do family interventions improve health?. J Fam Nurs.

[20] Dehbozorgi R, Shahriari M, Fereidooni-Moghadam M, Moghimi-Sarani E (2023). Family-centered collaborative care for patients with chronic mental illness: a systematic review. J Res Med Sci.

[21] Gilliss CL, Pan W, Davis LL (2019). Family involvement in adult chronic disease care: reviewing the systematic reviews. J Fam Nurs.

[22] Nur ABSS, Chua JYX, Shorey S (2023). Effectiveness of community-based family-focused interventions on family functioning among families of children with chronic health conditions: a systematic review and meta-analysis. Fam Process.

[23] Ostlund U, Persson C (2014). Examining family responses to family systems nursing interventions: an integrative review. J Fam Nurs.

[24] Park M, Giap TTT, Lee M, Jeong H, Jeong M, Go Y (2018). Patient- and family-centered care interventions for improving the quality of health care: a review of systematic reviews. Int J Nurs Stud.

[25] Seniwati T, Rustina Y, Nurhaeni N, Wanda D (2023). Patient and family-centered care for children: a concept analysis. Belitung Nurs J.

[26] Bell JM, Wright LM (2015). The Illness Beliefs Model: advancing practice knowledge about illness beliefs, family healing, and family interventions. J Fam Nurs.

[27] Azcárate-Cenoz Nerea, Canga-Armayor A, Alfaro-Díaz Cristina, Canga-Armayor N, Pueyo-Garrigues M, Esandi N (2024). Family-oriented therapeutic conversations: a systematic scoping review. J Fam Nurs.

[28] Preusse-Bleuler B (2021). Family-Centred Nursing Care: Textbook for Family-Assessment and Intervention, 3rd ed.

[29] Naef R, Kläusler-Troxler M, Ernst J, Huber S, Dinten-Schmid B, Karen T, Petry H (2020). Translating family systems care into neonatology practice: a mixed method study of practitioners' attitudes, practice skills and implementation experience. Int J Nurs Stud.

[30] Naef R, Meier Kaeppeli B, Lanter R, Petry H (2020). Implementing family systems care through an educational intervention with nurses and midwives in obstetrics and gynecological care: a mixed-methods evaluation. J Fam Nurs.

[31] Swiss Federal Office of Public Health (FOPH) (2020). Support Program. "Relief Offers for Caring Relatives 2017-2020".

[32] Zipfel N, Horreh B, Hulshof CTJ, de Boer AGEM, van der Burg-Vermeulen SJ (2022). The relationship between the living lab approach and successful implementation of healthcare innovations: an integrative review. BMJ Open.

[33] Archibald M, Wiebe S, Rieger K, Linton J, Woodgate R (2021). Protocol for a systematic review of living labs in healthcare. BMJ Open.

[34] Bell JM (2008). The Family Nursing Unit, University of Calgary: reflections on 25 years of clinical scholarship (1982-2007) and closure announcement. J Fam Nurs.

[35] Duhamel F, Dupuis F, Turcotte A, Martinez AM, Goudreau J (2015). Integrating the Illness Beliefs Model in clinical practice: a family systems nursing knowledge utilization model. J Fam Nurs.

[36] Benzein EG, Hagberg M, Saveman BI (2008). 'Being appropriately unusual': a challenge for nurses in health-promoting conversations with families. Nurs Inq.

[37] Hoffmann C (2015). Merging education, research, and care. Bull ZHAW Sch Health Sci.

[38] Pratt KJ, Sonney JT (2020). Fam Syst Health.

[39] Titchen A (2015). Action research: genesis, evolution and orientations. Int Pract Dev J.

[40] Creswell JW, Plano CVL (2018). Designing and Conducting Mixed Methods Research, 3rd ed.

[41] Yin R (2018). Case Study Research and Applications: Design and Methods, 6th ed.

[42] Creswell JW, Poth CN (2018). Qualitative Inquiry Research Design: Choosing Among Five Approaches.

[43] Sandelowski M (2011). "Casing" the research case study. Res Nurs Health.

[44] Thorne S (2016). Interpretative Description: Qualitative Research for Applied Practice.

[45] Vaismoradi M, Turunen H, Bondas T (2013). Content analysis and thematic analysis: implications for conducting a qualitative descriptive study. Nurs Health Sci.

[46] Sandelowski M (2010). What's in a name? Qualitative description revisited. Res Nurs Health.

[47] MAXQDA Software for qualitative data analysis, 1989-2024.

[48] Polit DF, Beck CT (2021). Nursing Research: Generating and Assessing Evidence for Nursing Practice, 11th ed.

[49] Kim H, Sefcik JS, Bradway C (2017). Characteristics of qualitative descriptive studies: a systematic review. Res Nurs Health.

[50] Malterud K, Siersma VD, Guassora AD (2016). Sample size in qualitative interview studies: guided by information power. Qual Health Res.

[51] Moriña A (2021). When people matter: the ethics of qualitative research in the health and social sciences. Health Soc Care Community.

[52] Rettke H, Pretto M, Spichiger E, Frei IA, Spirig R (2018). Using reflexive thinking to establish rigor in qualitative research. Nurs Res.

[53] Roberts D (2010). Vicarious learning: a review of the literature. Nurse Educ Pract.

[54] Ramsey R, Kaplan DM, Cross ES (2021). Watch and learn: the cognitive neuroscience of learning from others' actions. Trends Neurosci.

[55] Creswell JW, Creswell JD (2018). Research design: Qualitative, Quantitative, and Mixed Methods Approaches, 5th ed.

[56] Smith J, Ali P, Birks Y, Curtis P, Fairbrother H, Kirk S, Saltiel D, Thompson J, Swallow V (2020). Umbrella review of family-focused care interventions supporting families where a family member has a long-term condition. J Adv Nurs.

